# The Role of Aflatoxins in Hepatocellular Carcinoma

**DOI:** 10.5812/hepatmon.7238

**Published:** 2012-10-11

**Authors:** Hui Chen Wu, Regina Santella

**Affiliations:** 1 Department of Environmental Health Sciences, Mailman School of Public Health of Columbia University, New York, USA; 2 Herbert Irving Comprehensive Cancer Center, Columbia University Medical Center, New York, USA

**Keywords:** Aflatoxins, Aflatoxin-albumin Adducts, Hepatitis B Virus, Hepatocellular Carcinoma

## Abstract

**Context:**

Hepatocellular carcinoma (HCC) is one of the most common cancers in the world but with a striking geographical variation in incidence; most of the burden is in developing countries. This geographic variation in HCC incidence might be due to geographic differences in the prevalence of various etiological factors.

**Evidence Acquisition:**

Here, we review the epidemiological evidence linking dietary exposure to aflatoxin B1 (AFB1) and risk of HCC, possible interactions between AFB1 and hepatitis B virus (HBV) or polymorphisms of genes involved in AFB1-related metabolism as well as DNA repair.

**Results:**

Ecological, case-control and cohort studies that used various measures of aflatoxin exposure including dietary questionnaires, food surveys and biomarkers are summarized.

**Conclusions:**

Taken together, the data suggest that dietary exposure to aflatoxins is an important contributor to the high incidence of HCC in Asia and sub-Saharan Africa, where almost 82% of the cases occur.

## 1. Context

Hepatocellular carcinoma (HCC) is the fifth most common cancer in men and the seventh in women worldwide (1). Due to its poor prognosis, HCC is the third leading cause of cancer-related mortality. There is a striking geographical variation in the incidence of HCC and most of the burden is in developing countries, where over 80% of the cases occur (1). The regions of high incidence of HCC are Eastern and South-Eastern Asia and Middle and Western Africa (1). The geographic variation in HCC incidence might be due to geographic differences in the prevalence of various etiological factors, particularly chronic infection with hepatitis B and/or C virus, and dietary exposure to aflatoxins (2). Chronic hepatitis B Virus (HBV) infection prevalence ranges geographically from 0.2% to 20% (3). Approximately 45% of chronic HBV carriers live in highly endemic areas, such as Africa and the Asia-Pacific region where there is also a high incidence of HCC (3). Chronic HBV infection is believed to be responsible for 55% of HCC cases worldwide and 89% in regions where the virus is endemic or hyperendemic (4, 5). Although chronic HBV infection is the major risk factor for HCC, other environmental exposures such as drinking alcohol, tobacco smoking and aflatoxins have also been suggested to increase risk (6). Aflatoxins are naturally occurring mycotoxins produced by only a few Aspergillus species of which A. flavus and A. parasiticus are the most important; they live in hot and humidified conditions. Aflatoxins commonly contaminate foods such as peanuts, grain, legumes, and corn. They are carcinogenic in experimental animal models and aflatoxin B1 (AFB1) is the most potent hepatocacinogen (7). Once ingested, AFB1 is metabolized by the cytochrome P-450 system at the 8,9 -vinyl bond to produce an unstable reactive intermediate, AFB1-8,9-epoxide (8). This intermediate can bind covalently to DNA (9), forming AFB1-guanine adducts, and to protein, forming AFB1-albumin and other protein adducts (10, 11). The formation of AFB1-guanine adducts in hepatic DNA is critical for the carcinogenic effects of AFB1 in animals resulting in mutations in key genes (12). Assessing liver tumor tissues from regions with high exposure, a specific missense mutation at codon 249 in P53, AGG to AGT, leading to a substitution of arginine for serine (R249S), was frequently observed (13-15). Knowledge of the metabolism and toxicology of AFB1 has been used to develop biomarkers of exposure to study its carcinogenic effects (16, 17). These include measurement of aflatoxin metabolites in urine, DNA and protein adducts in blood and tissues and excised guanine adduct in urine. In addition to the formation of adducts, it is believed that AFB1 acts as a carcinogen by mechanisms that include the formation of reactive oxygen species leading to increased hepatic oxidative damage (18, 19). In our previously studies, we found that AFB1 exposure was positively associated with level of oxidative DNA damage in humans as measured by urinary 8-oxo-7, 8-dihydro-2′-guanine (20) as well as urinary 15-F2t-isoprostane, a marker of lipid peroxidation (21). Increased HCC risk was also associated with higher levels of these oxidative stress markers in prospective cohort (21). In the following, we review the epidemiological evidence linking dietary exposure to AFB1 and increased in risk of HCC, possible interactions between AFB1 and HBV or genetic polymorphisms of genes involved in AFB1-related metabolism as well as DNA repair.

## 2. Evidence Acquisition

### 2.1. Ecological Studies of Dietary Exposure to Aflatoxins and HCC Incidence/Mortality

Many ecological studies of HCC used food frequency questionnaires combined with food sampling surveys of AFB1 contamination to estimate dietary intake at the population rather than individual level in different parts of the world (Table 1). A study in the early 1970s measured AFB1 levels in major diet components from different parts of Uganda over one year and found that the frequency of AFB1 contamination was particularly high in provinces with high HCC incidence (22). Similar associations were reported in Swaziland (23) and Thailand (24). Data from Kenya quantifying daily intake based on assumed intakes of AFB1 contaminated food found those with higher average intakes were residents in areas with an elevated incidence of HCC (25). Pooling these observations (22-25) confirmed higher HCC incidence with higher levels of AFB1 consumption (26). Other ecological studies that determined AFB1 ingestion levels by collecting “food from the plate” consistently found significant correlations between calculated ingested daily dose and adult male incidence of HCC in different parts of Swaziland (27) and other countries in Southeastern Africa (28). That AFB1 is a cause of HCC in the U.S. was also suggested by comparing age-standardized HCC mortality rates and past dietary exposure to AFB1 in the Southeastern, Northern and Western regions where there was considerable geographic variation in the occurrence of HCC (29). Although HBV infection is also prevalent in countries with higher AFB1 exposure, none of these cross sectional correlations of AFB1 exposure and HCC risk was adjusted for prevalence of HBV infection (22-29). Examining levels of AFB1 in dietary samples over one year from 4 different geographic regions of Swaziland with a 5-fold range of HCC incidence, Peer et al. (30) reported HCC incidence was strongly associated with estimated levels of AFB1, while the proportion of HBV infection varied relatively little by geographic region. Similarly, when mortality rates were plotted against the estimated levels of exposure based on the regular collection and testing of sample foods consumed in Southern Guangxi, China over 6 years (1978-1984), a positive and almost perfectly linear relationship was observed between mean annual per capital consumption of AFB1 and HCC mortality rate for 1982-1986 across the communities (31).

**Table 1:** Ecological Study of Dietary Exposure to Aflatoxins and HCC

	Measure of AFB1 Exposure	Results	Comments
**Uganda, East Africa (1971) (22)**	Food sampling survey	Frequencies of AFB1 contamination high in areas with high incidence	No HBV prevalence data
**Swaziland, Southern Africa ( 1971) (23)**	Food sampling survey	Higher risk in areas with highest frequency of AFB1 contamination	No HBV prevalence data
**Thailand, Southeast Asia (1972) (24)**	Food sampling survey	Higher risk in areas with highest frequency of AFB1 contamination	No HBV prevalence data
**Kenya, East Africa (1973) (25)**	Daily AFB1 intake calculated from AFB1-comtaminated food sampling from markets; assumed 2kg intake/day and 70kg average adult body weight	Positive association with AFB1 ingestion levels and HCC incidence rate	No HBV prevalence data
**International comparison: Uganda, Swaziland, Thai**land and Kenya (1975) (26) a****	Data from references (22-25)	Countries with higher HCC risk had highest levels of AFB1 contamination	International comparison; no HBV prevalence data
**Swaziland, Southern Africa (1976) (27)**	Sampling food from the plate over one year	Positive correlation between consumption of AFB1 during 1972-1973 and HCC incidence rate for 1964-1968.	No HBV prevalence data
**US (1985) (28)**	Food sampling survey	Expected average daily ingestion of AFB1 positively associated with HCC mortality	No HBV prevalence data
**Mozambique and Transkei, Southeastern Africa (1983) (29)**	Sampling food from the plate over four years	Mean AFB1 dietary intake significantly associated with HCC rates	No HBV prevalence data
**Swaziland, Southern Africa (1987) (30)**	Sampling food from the plate over one year	AFB1 , not HBV, associated with HCC incidence	Blood bank HBV prevalence data; age distribution heavily skewed towards young-adults
**Kenya, East Africa (1989) (31)**	Prevalence of urinary excretion of AFB1-Guanine Adduct	Association of AFB1-Guanine adduct with HCC limited to one racial group	No interaction between HBV infection and AFB1 exposure on HCC risk
**Guangxi, China (1987) (32)**	Food sampling survey	Positive linear relationship between HCC rate and AFB1 exposure	Regional prevalence of HBV not associated with HCC rate; individual with chronic HBV infection had about 39 fold increased risk of HCC than individuals with no HBV infection
**Nation-wide, China (1990) (33)**	Urinary AFB1 metabolites	HCC mortality was unrelated to urinary AFB1 metabolites	Adjusted for prevalence of HBV
**Taiwan (1993) (34)**	Urinary AFB1 metabolites	There was a significant association between the marker of AFB1 exposure and the background rate of HCC mortality	Association of AFB1 exposure with HCC stronger among females with than without HBV infection but not among males

Abbreviations: AFB1, aflatoxin B1; HBV, hepatitis B virus; HCC, hepatocellular carcinoma.

^a^Correlated AFB1 levels and incidences of HCC of the first 4 studies.

Later studies monitored AFB1 exposure by measuring urinary excretion of AFB1 metabolites and released DNA adducts (32, 33). The first study measuring urinary AFB1-DNA adducts in Kenya found a moderate degree of correlation between AFB1 exposure and HCC only when the study was limited to certain ethnic groups and no synergistic effect of exposure and HBV infection on HCC risk (32). Another cross-sectional study conducted throughout China did not find an association between urinary AFB1 metabolites and HCC mortality (33). However, urine samples were collected in 1983, while data on HCC mortality was between 1973 and 1975. Analysis of the relationship between levels of urinary AFB1 metabolites and HCC mortality will be valid only if exposure levels are stable over time. Despite of the lack of association in these two studies, our cross-sectional survey of 250 residents from 8 areas of Taiwan with a 4-fold variation in HCC mortality found a positive association between urinary AFB1 metabolites and mortality at the township level and the association was stronger in females with chronic HBV infection than in those uninfected (34). Overall, many ecological studies have found a significant association between dietary AFB1 intake, estimated from contaminated foods, and HCC incidence in different populations, while data on the cross-sectional relationship between urinary AFB1 markers and HCC incidence are discordant. There are some methodological issues that need to be considered in these ecological studies. First, many studies were conducted in high HBV endemic regions and did not consider the effect of HBV on HCC risk. Thus, it is uncertain how much of the association could be explained by HBV carrier state (33). Second, the reliability of data on HCC incidence, and prevalence of HBV is limited since cancer registries and HBV screening were not nation-wide in these areas (33). Third, the estimate of AFB1 exposure was based on sampling some particular foods in the market or from households; seasonal variation that might affect the levels of contamination was not considered. Moreover, measuring AFB1 intake by food analysis is subject to its highly erratic distribution in different foods (35). The lack of individual-level information is a limitation of ecological studies known as the “ecological fallacy” meaning an association observed between variables on an aggregate level does not necessarily represent the association that exists at the individual level. Thus, one cannot necessarily infer the same relationship from the group level to the individual level. However, ecological studies can be done quickly and inexpensively and have provided useful information on the association of AFB1 intake and HCC risk.

### 2.2. Cases-Control Studies of Dietary Exposure to Aflatoxins and HCC Risk

Further studies confirming an etiological role for AFB1 have mainly been conducted in Asia comparing levels of AFB1 exposure between HCC cases and controls (Table 2). Early case-controls studies estimated individual levels of dietary AFB1 consumption retrospectively from dietary questionnaires data (36-41). A study conducted in the Philippines compared dietary intakes of HCC cases with age- and sex-matched controls (36). By using dietary recall, the frequency and amounts of food items consumed were used to calculate units of AFB1 load per day. These calculations revealed that the mean AFB1 load of HCC cases was 4.5 times higher than that of controls. Although this study did not have HBV infection data, AFB1 and alcohol, when consumed concurrently, were found to act synergistically in the development of HCC in males (36). A similar case-control study carried out in Henan, China reported odds ratios (ORs) of 13.5 (95% confidence interval (CI) = 3.7-51.2) for peanut and peanut oil consumption and of 19.4 (95%CI= 3.7-103.0) for corn consumption (41). In contrast, based on questionnaire data, consumption of more food thought most likely to be contaminated with AFB1, such as peanuts, had a no significant positive association with risk of HCC in Hong Kong (37) and Taiwan (38, 40).

**Table 2:** Case-Control Study of Aflatoxins and HCC Risk

	Cases, No.	Controls, No.	Measure of AFB1 Exposure	Result	Adjusted for HBV Status
**Philippines ** **(1982) (36)**	90	90	Dietary questionnaire	Synergistic interaction between AFB1 and alcohol intake on HCC risk	No
**Hong Kong ** **(1982) (37)**	107 (95 M, 12 F)	107 (94 M, 13 F) Hospital-based	Dietary questionnaire	No association of risk with consumption with AFB1-contaminated foods	Yes
**Taiwan ** **(1988) (38)**	131 (121 M, 10 F)	207 (195 M, 12 F) Hospital-based	Peanut consumption	No association between peanut consumption and risk	Yes
**Thailand ** **(1991) (39)**	65 (47 M, 18 F)	65 (47 M, 18 F) Hospital-based	Peanut consumption; AFB1-albumin adducts	No increase in risk with AFB1 intake, as estimated by consumption of contaminated foods, or by measuring serum AFB1-albumin adducts	Yes
**Taiwan ** **(1991) (40)**	200 M	200 M Community-based	Peanut consumption	Frequency of peanut consumption not associated with risk	No
**Henan, China (1998) (41)**	152 (136 M, 16 )	115 (99 M, 16 F) Hospital based	Dietary questionnaire	Positive association between risk and peanut and/or corn consumption	Yes
**Guangxi, China a (2008) (42)**	491 (362 M, 129 F)	862 (641 M, 221 F) Hospital-based	Leukocyte AFB1-DNA adducts	Dose relationship between AFB1-DNA adduct and HCC risk,	Yes
**Guangxi, China (2009) (43)**	618 (448 M,170 F)	712(540 M, 172 F)	Years of living in AFB1 exposure area; leukocyte AFB1-DNA adduct	Dose relationship between risk and AFB1 as measured by years of residence and AFB1-DNA adduct	Yes
**Guangxi, China (2011) (44)**	1156	1402	Years of living in AFB1 exposure area; leukocyte AFB1-DNA adduct	Dose relationship between risk and AFB1 as measured by year of resident and AFB1-DNA adduct	Yes
**Guangxi, China (2010) (45)**	348 (263 M, 85 F)	597 (459 M, 138 F)	Years of living in AFB1 exposure area; leukocyte AFB1-DNA adduct	Dose relationship between risk and AFB1 as measured by year of resident and AFB1-DNA adduct	Yes
**Southern Guangxi, China (2011) (46)**	60 (54 M, 6 F)	120 (108 M, 12 F) Population-based	Serum AFB1-lysine adduct	No significant difference in levels of AFB1-lysine adduct	Yes
**India ** **(2010) (47)**	266 (211 M, 55 F)	251 (176 M, 75 F)	Food sampling from each meal for each subject over 1 week; urinary AFB1-N7-Gua	No main effect of AFB1 on risk; combined effect of AFB1 and HBV was significant	Yes

Abbreviations: AFB1, aflatoxin B1; F, females; HBV, hepatitis B virus; HCC, hepatocellular carcinoma; M, males.

^a^Same study population with increasing recruitment over time.

Other case-control studies estimated AFB1 exposure using biomarkers (39, 42-46). In one study Thailand, two approaches were used to estimate exposure, intake estimated by consumption of possibly contaminated foods and measurement of AFB1-albumin adducts in serum (39). No increase in risk was found with AFB1 exposure by either measure. In a population-based case–control study conducted in Southern Guangxi, China, although levels of serum AFB1-lysine adduct were higher in cases than controls, there was no significant association with HCC risk (45). Another series of studies also in Gugngxi, China found a dose-response relationship between HCC risk and levels of AFB1-DNA adducts in leukocytes and AFB1-exposure years, as measured by years living in a high AFB1 exposure region (42-44, 47). Moreover, there was a strong synergistic interaction between AFB1 exposure and genetic polymorphisms in genes involved in the repair of DNA double strand breaks (i.e. XRCC3 (42) and XRCC7 (44)) or nucleotide excision repair (i.e. XPD (43) and XPC (47)).

In summary, in case-control studies, dietary data generally do not show an increase in risk of HCC associated with ingestion of AFB1-contaminated foods, although conflicting data were reported. As mentioned above, dietary questionnaire data are inadequate to measure AFB1 intake because the content of individual foods can vary widely due to geographic and seasonal variations. This method also suffers from severe recall bias. Thus, the weak association may result from the inadequacy of the questionnaire in measuring AFB1 exposure. Biomarkers of AFB1 exposure that measure adducts quantitate the biologically effective dose at the individual level. However, subjects were selected on the basis of disease status. It is unclear whether cancer status affects the formation of biomarkers, and the association of biomarkers and HCC risk. The advantages of case-control studies are their ability to assess the relationship of AFB1 exposure and HCC risk at the individual level, and to adjust for potential confounder such as HBV infection. Although the studies also suggest an interaction effect of AFB1 exposure and other risk factors, the conclusions are limited because of relatively small sizes.

### 2.3. Cohort Studies of Dietary Exposure to Aflatoxins and HCC Risk

A more promising and valid approach to evaluating the role of AFB1 is through prospective studies, where levels of AFB1 biomarkers are measured in blood/urine specimens collected years before diagnosis. To date, three major cohort studies have used this study design to address the relationship of AFB1 exposure and HBV infection to HCC incidence (48-55). The first case-control study nested in a cohort of 18,244 men was conducted in Shanghai, People’s Republic of China (48, 49). After nearly 35,299 person years of follow-up, cases (n = 22) were matched to controls for age and area of residence. The relative risk (RR) of HCC for the presence of AFB1 metabolites including unmetabolized aflatoxins, hydroxylated and demethylated metabolites, and AFB1-N7-guanine adducts, after adjusting for other factors including hepatitis B virus surface antigen status, was 3.8 (95% CI = 1.2-12.2). A strong interaction between AFB1 and HBV was also reported with an RR of 60.1 (6.4-561.8) although there were only 7 cases and 2 controls who were both HBsAg positive and positive for the presence of AFB1 metabolites (48). A subsequent follow-up study from this cohort with a total of 50 cases showed that the presence of any urinary AFB1 biomarker significantly predicted liver cancer (RR = 5.0; 95% CI, 2.1-11.8) (49). The synergistic interaction between AFB1 exposure and HBV infection on HCC risk was again observed (49). However, using a dietary questionnaire and food survey to assess individual AFB1 intake, no association was found (49). Our Cancer Screaning Project (CSP) cohort recruited resident of age of 30 to 65 living in several rural townships of Taiwan where HBV infection is hyperendemic (50, 53, 55, 56). Preliminary results from this prospective cohort, limited to resents living on the Penghu Island who have the highest HCC mortality and AFB1 exposure in Taiwan, demonstrated a 5.5-fold increased risk of HCC with detectable AFB1-albumin adducts (50). The entire cohort includes 12,024 males and 13,594 females, and 56 of these individuals developed HCC during the first 3-4 years of follow-up and 230 HCC cases were identified after 12 years (53, 55). Analysis of urine and albumin samples banked 1 to 12 years before diagnosis gave a relative risk of 1.5 (95% CI = 1.0-2.6) for AFB1-albumin adducts and 1.8 (95%CI = 1.2-2.6) for AFB1 metabolites (55). In the recent larger study, we also observed a 4-fold increased risk of HCC for HBsAg negative individuals with higher urinary AFB1 metabolites. In the first smaller study, we reported a 111-fold increased risk of HCC among HBsAg carriers with high urinary AFB1 metabolites and 70-fold for detectable AFB1-albumin adducts compared with those with low/nondetectable levels and negative for HBsAg (53) In the later study, with a much larger sample size, the effect of combined AFB1 exposure and HBV infection is more consistent with an additive than a multiplicative model (55). To date, there are no other data on the long-term combined effect of AFB1 and HBV on HCC risk.

Another study we conducted in Taiwan among 4841 male government workers with chronic HBV infection provided additional evidence for the role of AFB1 intake in development of HCC (51, 52). With 5 years of follow up, we observed a dose-response relationship between HCC and serum level of AFB1-albumin adducts (51) and urinary AFB1 metabolites (52) among those who had null genotypes of GSTM1 and T1 but not among those who had non-null genotypes. Consistent results were found in our CSP cohort when we restricted analysis to HBV carriers (56). In a cohort of Chinese men with chronic HBV followed for 10 years, the relative risk of HCC was significantly increased in subjects with detectable afLatoxins M1 (AFM1) levels (54). This prospective cohort study demonstrated that detectable urinary AFM1 levels above 3.6ng/L were associated with increased risk of HCC in male HBsAg carriers with chronic hepatitis (54). No genotype data were available in this study (Table 3). 

**Table 3:** Nested Case-Control Study of Aflatoxins and Hepatocellular Carcinoma Risk

	Cohort, No.	Follow up, y	Cases, No.	Controls, No.	AFB1 Measurement	Individual Effect of AFB1 RR (95%CI)	Individual Effect of HBV RR (95%CI)	Combined Effect of AFB1 and HBV RR (95%CI)
**Shanghai, China (1992) (48)**	18, 244 males aged of 45 to 64							
		1	22	140	Urinary AFB1 metabolites	3.8 (1.2-12.2) for present of urinary AFB1	8.5 (2.8-26.3)	60.1 (6.4-561.8)
		3	50	267	Urinary AFB1 metabolites; dietary questionnaire	5.0 (2.1-11.8) for presence of urinary AFB1; no association between dietary AFB1 exposure and HCC risk	7.3 (2.2-4.2)	59.4 (16.6-212.0)
**Government Employees, Tai**wan (1994) (49)****	4841 HBV carriers males aged of 30 to 65							
		4.7	32	73	AFB1-albumin adducts	3.8 (1.0-14.5) for high AFB1-albumin adduct; effect mainly among those with null genotypes of GSTM1 or GSTT1		
		4.7	43	43	AFB1-albumin adducts; urinary AFB1 metabolites	6.0 (1.2-29.0) for high urinary AFM1; effect mainly among those with GSTM1 null genotype		
**Cancer Screen**ing Project (CSP), Taiwan (1996) (50)****								
	6487 residents aged of 30 to 65	1	20	86	AFB1-albumin adducts	5.5 (1.2-24.5)	129.4 (25.4-659.2)	
	12,024 males and 13,594 females aged of 30 to 65	3	56	220	AFB1-albumin adducts;Urinary AFB1 metabolites	3.8 (1.1-12.8) for high *vs.* low urinary AFB1 metabolites; 1.6 (0.4-5.5) for detectable *vs. *nondetectable AFB1-albumin adducts Among HBV carriers: 5.5 (1.3-23.4) for high *vs.* low urinary AFB1 metabolites; 2.8 (1.3-23.4) for detectable* vs.* nondetectable AFB1-albumin adducts	45.5 (13.8-149.7)	111.9 (13.8-905.0) for high vs. low urinary AFB1 metabolites; 70.0 (118.8-415.4) for detectable* vs.* nondetectable AFB1-albumin adducts
	among HBV carriers	5	79	149	AFB1-Albumin adducts	2.0 (1.1-3.7 for detectable *vs.* nondetectable AFB1-albumin adducts; effect mainly among those with null genotypes of GSTM1 or GSTT1		
	12024 males and 13594 females aged of 30 to 65	12	230	1052	AFB1-Albumin adducts; urinary AFB1 metabolites	1.8 (1.2-2.6) for high *vs.* low urinary AFB1 metabolites; 1.5 (1.2-2.6) for high vs. low AFB1-albumin adducts Among HBV noncarriers: 4.3 (1.4-12.9) for high *vs.* low urinary AFB1 metabolites	7.5 (5.1-10.9)	15.1 (7.8-29.3) for high *vs.* low urinary AFB1 metabolites; 10.4 (5.7-18.8) for high *vs.* low AFB1-albumin adducts; an additive effect of HBV and AFB1 exposure on HCC risk
**Qidong, China (1996) (51)**								
	145 males with HBV-related hepatitis	10	22		AFM1	3.3 (1.2-9.0) for high *vs. *low urinary AFM1		

Abbreviations: AFB, aflatoxin; HBV, hepatitis B virus; HCC, hepatocellular carcinoma; RR, Relative Risk

## 3. Results

Overall, prospective studies have shown a strong association between biological markers of AFB1 exposure in serum or urine and risk of subsequent HCC. The interaction between AFB1 exposure and HBV infection on HCC risk was replicated in different cohorts. However, earlier studies including ours suggested a synergistic effect, while in our recently follow up, an additive interaction model was suggested. The primary reason for these discrepant results may be the small sample sizes in earlier studies. Genetic variation in metabolic enzymes as well as DNA repair might play a role in the risk of AFB1-related HCC; however, no large scale study has comprehensively examined the effect of genotype on the association between AFB1 exposure and HCC risk.

## 4. Conclusions

Many ecological studies of AFB1-contaminated foods consistently suggest a role for this carcinogen in HCC. Although earlier data from case-control studies are conflicting, strong evidence for an AFB1-HCC link was subsequently provided by studies based on the detection of AFB1 markers in biospecimens collected years before diagnosis. All ecological studies of dietary AFB1 and HCC were conducted in regions where HBV infection is hyperendemic but only two studies examined the roles of AFB1 exposure and HBV infection in explaining the geographic variation of HCC incidence. However, no attempt was made to evaluate a possible interactive effect between these two risk factors. Case-control studies also did not evaluate interactions. The development of biomarkers for measuring AFB1 metabolites and AFB1-DNA and albumin adducts resulted in more accurate and reliable quantification of dietary AFB1 exposure. All cohort studies measuring biomarkers of AFB1 consistently demonstrated that high levels of exposure increased risk of developing HCC later in life. All cohort studies suggest an interaction effect between AFB1 and HBV infection. A striking multiplicative effect was suggested in the earlier cohort studies with small numbers of HCC cases and short-term follow-up, including ours. In contrast, our more recent data suggested that the combined effect of AFB1 exposure and HBV infection is more consistent with an additive than a multiplicative model. A quantitative cancer risk assessment estimated that aflatoxin may play a causative role in 4.6–28.2% of all global HCC cases (57). Although the etiological role of AFB1 exposure on HCC has been investigated for 50 years, data on the interaction of hepatitis B/C virus infection, genetic susceptibility and life style are limited. National HBV vaccination programs have been conducted in many counties, particularly in regions where AFB1 exposure and HBV prevalence are high (58). This will likely increase the importance of understanding the burden of other HCC risk factors. While some case-controls studies suggested an interaction effect of AFB1 exposure and genetic susceptibility and/or alcohol consumption on HCC risk, additional large scale population studies are needed to fully understand the carcinogenic mechanism of AFB1.
